# Fatigue in young adults with juvenile idiopathic arthritis 18 years after disease onset: data from the prospective Nordic JIA cohort

**DOI:** 10.1186/s12969-021-00499-0

**Published:** 2021-03-18

**Authors:** Ellen Dalen Arnstad, Mia Glerup, Veronika Rypdal, Suvi Peltoniemi, Anders Fasth, Susan Nielsen, Marek Zak, Kristiina Aalto, Lillemor Berntson, Ellen Nordal, Troels Herlin, Pål Richard Romundstad, Marite Rygg, on behalf of the Nordic Study Group of Pediatric Rheumatology (NoSPeR), Gudmund Marhaug, Boel Anderson-Gäre, Freddy Karup Pedersen, Pekka Lahdenne, Pirkko Pelkonen

**Affiliations:** 1 Department of Pediatrics, Levanger Hospital, Nord-Trøndelag Hospital Trust, Pb 333, 7601 Levanger, Norway; 2 Department of Clinical and Molecular Medicine, NTNU - Norwegian University of Science and Technology, Trondheim, Norway; 3 Department of Pediatrics, Aarhus University Hospital, Aarhus, Denmark; 4 Department of Pediatrics, University Hospital of North Norway and Department of Clinical Medicine, UIT the Arctic University of Norway, Tromsø, Norway; 5 New Children’s Hospital, Helsinki University Hospital, Pediatric Research Center, University of Helsinki, Helsinki, Finland; 6 Department of Pediatrics, Institute of Clinical Sciences, Sahlgrenska Academy, University of Gothenburg, Gothenburg, Sweden; 7 Department of Pediatrics, Rigshospitalet Copenhagen University Hospital, Copenhagen, Denmark; 8 Department of Women’s and Children’s Health, Uppsala University, Uppsala, Sweden; 9 Department of Public Health and Nursing, NTNU - Norwegian University of Science and Technology, Trondheim, Norway; 10 Department of Pediatrics, St. Olavs Hospital, Trondheim, Norway

**Keywords:** Juvenile idiopathic arthritis (JIA), Fatigue, Patient-reported outcomes, Health-related quality of life (HRQoL), Young adults, Long-term outcomes

## Abstract

**Background:**

To study fatigue in young adults with juvenile idiopathic arthritis (JIA) 18 years after disease onset, and to compare with controls.

**Methods:**

Consecutive children with onset of JIA between 1997 and 2000, from geographically defined areas of Norway, Sweden, Denmark and Finland were followed for 18 years in a close to population-based prospective cohort study. Clinical features, demographic and patient-reported data were collected. Inclusion criteria in the present study were a baseline visit 6 months after disease onset, followed by an 18-year follow-up with available self-reported fatigue score (Fatigue Severity Scale (FSS), 1–7). Severe fatigue was defined as FSS ≥4. For comparison, Norwegian age and sex matched controls were used.

**Results:**

Among 377 young adults with JIA, 26% reported severe fatigue, compared to 12% among controls. We found higher burden of fatigue among participants with sleep problems, pain, poor health, reduced participation in school/work, physical disability, active disease, or use of disease-modifying anti-rheumatic drugs (DMARDs)/biologics/systemic steroids. In contrast, participants without these challenges, had fatigue scores similar to controls. Active disease assessed at all three time points (baseline, 8-year and 18-year follow-up) was associated with higher mean fatigue score and higher percentage of severe fatigue compared to disease courses characterized by periods of inactive disease. Predictors of fatigue at the 18-year follow-up were female sex and diagnostic delay of ≥6 months at baseline, and also pain, self-reported poor health, active disease, and previous/ongoing use of DMARDs/biologics at 8 years.

**Conclusions:**

Fatigue is a prominent symptom in young adults with JIA, with higher fatigue burden among participants with poor sleep, pain, self-reported health problems, active disease, or use of DMARDs/biologics. Participants without these challenges have results similar to controls. Patient- and physician-reported variables at baseline and during disease course predicted fatigue at 18-year follow-up.

**Supplementary Information:**

The online version contains supplementary material available at 10.1186/s12969-021-00499-0.

## Background

Juvenile idiopathic arthritis (JIA) is a heterogeneous chronic childhood disease with onset before 16 years of age. JIA is the most common rheumatic disease among children with incidence rates in the Nordic countries of 12.8–23/100,000 children [1–3]. Despite efficient modern treatment, including biologics, many patients with JIA experience reduced health-related quality of life (HRQoL) [4–6], and suffer from pain, physical disability and reduced participation in school and leisure activities in a long-term perspective [6].

There is no uniform definition of fatigue, but it is often referred to as “*a persistent, overwhelming sense of tiredness, weakness or exhaustion, resulting in a decreased capacity of physical and/or mental work and is unrelieved by sleep or rest*” [7]. Fatigue is a complex interplay between many factors, even though the etiology of fatigue is still unknown.

Fatigue is a disabling symptom in a variety of chronic disorders, and a frequent complaint in both JIA and rheumatoid arthritis (RA) [8, 9]. Fatigue is reported by 60–76% in JIA [4, 10], and 62–98% in RA [11, 12], and leads to severe consequences in several life domains affecting every-day functioning. In a systematic review from 2016, Armbrust et al. concluded that the literature on fatigue in JIA is sparse, especially with fatigue as the main focus. Some JIA studies have shown an association between fatigue, sleep disturbances, pain and HRQoL, but an inconsistent relationship between fatigue and disease activity [7, 8, 13, 14]. The association with reduced quality of life is well documented in RA [9, 12, 15]. Both adults with JIA and RA have stated fatigue as one of the most important outcomes of their disease [16, 17].

The European League Against Rheumatism and the American College of Rheumatology have, in collaboration, named fatigue as an outcome of particular importance in RA, which should be routinely assessed in clinical trials [18]. This suggests that fatigue may also be an important outcome in JIA. Over the last decade, fatigue has become an issue of interest among clinicians and an increasing research priority in adult rheumatology, but few studies focus on fatigue in pediatric rheumatology [7]. Knowledge of fatigue in long-term follow-up of JIA, is limited.

The aim of this study was to reduce the existing knowledge-gap on fatigue as an important long-term outcome in JIA, by looking at the prevalence and severity of fatigue in young adults with JIA followed prospectively for approximately 18 years after disease onset. We also aimed to explore the association with sleep disturbances, pain, self-reported health problems and disease activity, and compare the results to a control group.

## Methods

### Patients

The Nordic JIA cohort is a close to population-based multicenter cohort study. Consecutive children diagnosed with JIA between January 1, 1997 to June 30, 2000, from geographically defined areas in Norway, Sweden, Denmark and Finland, were prospectively included. To ensure the referral of all eligible candidates, letters were distributed to all general practitioners and specialists in rheumatology/pediatrics/orthopedics in the catchment areas. The baseline visit was scheduled to take place 6 months after disease onset, with regular follow-up thereafter, including an extended 8-year follow-up in 2005–2008 [19]. A detailed description of patient enrolment and data collection has previously been published [1, 19]. JIA categories were determined in accordance with the International League of Associations for Rheumatology (ILAR) classification criteria [20].

In 2014–2017, all 510 previously included participants were invited to participate in a follow-up study 17.5 (±1.7) years (mean (±SD)) after disease onset, later termed “the 18-year follow-up” [21]. Ten out of 510 were re-included at 18-year follow-up, because of incorrect exclusion after the baseline visit. In the present study, participants were included if they had at least a baseline visit and participated in the 18-year study with available self-reported fatigue scores.

### Data collection

The 18-year follow-up was composed of a study visit with clinical examination, including a full joint examination performed by experienced pediatric rheumatologists to explore whether the participants had active joints and/or restricted joints. In addition, a temporo-mandibular joint (TMJ) examination by a dentist, and an eye examination by an ophthalmologist were performed [22, 23]. We also registered ongoing and previous medication, disease status and damage, blood tests, self-reported questionnaires on health and HRQoL, including fatigue and sleep questionnaires and participation in school/work. If they were unable to attend the study visit, participants were invited to participate in a standardized telephone interview and to fill in self-reported questionnaires. The baseline and 8-year follow-up visits included data from clinical examinations, information about disease activity, medication and blood tests, and in addition results from self-reported questionnaires.

### Controls

Controls from Central Norway were randomly selected from the National Population Register of Norway. Eligible controls were matched by age and sex to the Norwegian participants from Central Norway, residing in both urban and rural areas. Invitations were sent asking for participation if they had no cancer, rheumatic or autoimmune diseases.

### Measures

At the 18-year follow-up, self-reported fatigue during the previous 2 weeks was measured with the validated Fatigue Severity Scale (FSS) available in all the Nordic languages. FSS comprises 9 items covering physical, social and cognitive effects of fatigue, giving a global score of 1–7 (1 = lowest fatigue, 7 = highest fatigue) [24, 25]. Severe fatigue was defined as FSS ≥4, in line with others [24, 26]. The FSS also includes a 21-numbered circle visual analogue scale (VAS) to measure fatigue severity. Sleep quality during the previous month was measured with the validated Pittsburgh Sleep Quality Index (PSQI) consisting of seven components: sleep duration, sleep disturbance, sleep latency, daytime dysfunction due to sleepiness, sleep efficiency, sleep quality and sleep medication, giving a global score of 0–21 (0 = best sleep, 21 = worst sleep). Poor sleep was defined as PSQI > 5, according to the form administration instruction of PSQI [27]. Self-reported, disease-related pain was measured on a 21-numbered circle VAS (0 = no pain, 10 = maximum pain) [28]. HRQoL was assessed with the generic multidimensional Medical Outcomes Study 36-Item Short-Form Health Survey (SF-36) yielding a physical/mental component summary (PCS/MCS) score (0–100, 0 = worst, 100 = best) [29]. Poor health was defined as PCS/MCS < 40 and better health ≥40, based on the United States general population’s average score of 50 with standard deviation (SD) of 10 [30]. Validated Norwegian, Swedish and Danish versions of SF-36 were used. The Finnish SF-36 version has previously been validated against the general Finnish population [31]. The validated disease-specific Health Assessment Questionnaire (HAQ) (0 = no difficulty, 3 = unable to do) was used to assess physical self-reported disability, dichotomized into =0 (no disability), or > 0 (disability) [19, 32, 33]. We used the American College of Rheumatology (ACR) provisional criteria for defining clinical inactive disease, which includes patient-reported morning stiffness ≤ 15 min, physician’s VAS global disease activity =0, normal erythrocyte sedimentation rate (ESR), no active uveitis and no fever, rash, serositis, splenomegaly or generalized lymphadenopathy attributed to JIA [34]. Remission was defined according to Wallace’s preliminary criteria; remission off medication was achieved if the participant had sustained inactive disease off medication for minimum 12 months, and remission on medication if the participant had sustained inactive disease on medication for minimum 6 months [35]. Clinical disease activity was measured with the juvenile arthritis disease activity score (JADAS71) (0–101, inactive disease ≤1) [36].

At the 8-year visit, the functional disability was measured with the generic Child Health Questionnaire (CHQ), with a physical/psychosocial summary score (PhS/PsS) (0–100, 0 = worst, mean 50 ± 10) [37], and the disease-specific Childhood HAQ (CHAQ) if age < 18 years.

### Statistical analysis

To summarize clinical characteristics, we used descriptive statistics with mean and SD/median and 1st-3rd interquartile ranges (IQR) for continuous variables and absolute frequencies and percentages for categorial variables. To estimate the odds ratio (OR) for severe fatigue with 95% confidence interval (CI), we used multivariable logistic regression analyses adjusted for age and sex. Statistical analyses were carried out using STATA version 16, software (STATA Corp., College Station, Texas, USA).

## Results

Of 510 eligible participants included from four Nordic countries, 434 (85%) participated in the 18-year follow-up, 329 (76%) with a clinical visit and 105 (24%) with a standardized telephone interview and questionnaires [21]. Eighty-seven percent of the participants (*n* = 377) completed the Fatigue Severity Scale (FSS) and were included in the present study, and of these 16% (*n* = 61) took part in a telephone interview. At the 18-year follow-up, median disease duration of included participants was 17.5 years, median age at follow-up was 23.3 years, median numbers of study visits were 6, 72% were female, 46% had oligoarticular disease, 42% were in remission off medication, and 31% used disease-modifying anti-rheumatic drugs (DMARDs) and/or biologics (Table 1).

**Table 1:** Clinical characteristics of the Nordic JIA study population

Characteristics	Total no. assessed	Values
Female sex, no. (%)	377	271 (72)
Age at disease onset, years, median (IQR)	377	5.6 (2.6–9.7)
Age at 18-year follow-up, years, median (IQR)	377	23.3 (20.2–27.1)
Disease duration at 18-year follow-up, years, median (IQR)	375	17.5 (16.8–18.3)
Oligoarticular JIA at onset, no. (%)	377	201 (53)
Oligoarticular^a^ JIA at 18-year follow-up, no. (%)	377	175 (46)
VAS pain at 18-year follow-up, mean (±SD)	370	1.9 (±2.4)
SF-36 PCS at 18-year follow-up, mean (±SD)	377	51.5 (±9.7)
SF-36 MCS at 18-year follow-up, mean (±SD)	377	49.1 (±11.3)
HAQ at 18-year follow-up, mean (±SD)	370	0.2 (±0.4)
Not in remission^b^ at 18-year follow-up (visits), no. (%)	308	196 (63)
Not in remission^b^ at 18-year follow-up (visits/telephone^c^), no. (%)	369	215 (58)
DMARDs and/or biologics ongoing at 18-year follow-up, no. (%)	377	118 (31)
DMARDs and/or biologics ever during disease course, no. (%)	377	239 (63)

*JIA* juvenile idiopathic arthritis, *no.* numbers, *IQR* interquartile range, 1st-3rd, *SD* standard deviation, *VAS pain* self-reported pain measured on a 21-numbered circle visual analogue scale (0 = no pain, 10 = maximum pain), *SF-36* Short-form 36 Health Status Questionnaire, 0–100 (< 40 poor health), *PCS* physical component summary, *MCS* mental component summary, *HAQ* Health Assessment Questionnaire, 0–3 (0 = lowest, 3 = highest), *DMARDs* disease-modifying anti-rheumatic drugs, *biologics* biologic drugs

^a^Persistent (no. =98) and extended (no. =77) oligoarticular disease

^b^Not in remission off medication according to the definition by Wallace et al.

^c^61 participated only in a telephone interview

When comparing the 76 individuals lost to follow-up to the 434 participants at the 18-year follow-up, they did not differ in age at onset, JIA category, sex or number of active joints during the first 6 months after disease onset. Participants excluded from the present study due to missing fatigue scores (*n* = 57) did not differ with respect to JIA category, age at onset, and age at follow-up, but there were more males (48% versus 28%) and more participants in remission off medication at the 18-year follow-up visit (59% versus 42%).

Of 265 invited controls, 136 did not answer, 3 refused to participate, 13 were excluded because they had moved outside Central Norway, and 3 were excluded due to illness. In the control group 110 participated, but one was later excluded due to possible rheumatic disease. The final control group consisted of 109 participants, 72% were female, and median age was 23.1 (IQR 20.0–26.6) years. The invited controls who did not answer or refused to participate, did not differ in age or sex, compared to the controls who participated.

### Fatigue scores

Mean (±SD) fatigue score was 3.2 (±1.5) among participants with JIA, and 2.8 (±1.1) (*p* = 0.06) among the Norwegian controls (Table 2 and Supplementary Figure S1). Severe fatigue defined as FSS ≥4 was reported by 26% of participants with JIA compared to 12% of controls (*p* = 0.002) (Table 2). We found only small differences in fatigue scores according to JIA categories (Supplementary Table S1). Further, we found no significant difference in mean (±SD) fatigue scores (3.1 (±1.3) versus 3.2 (±1.5), *p* = 0.7) or in the amount of severe fatigue (20% versus 28%, *p* = 0.1) between participants with telephone interview, compared to those with a clinical visit.

**Table 2:** Fatigue in the Nordic JIA cohort according to clinical characteristics at 18-year follow-up

			Severe fatigue^b^					
	No. assessed	Fatigue^a^ mean ±SD	No. (%)	OR (95% CI) crude	*p*-value	OR (95% CI) adjusted^c^	*p*-value	VAS fatigue^d^ mean ±SD
Norwegian controls	109	2.8 ±1.1	13 (12)	1.0 (ref.)	–	1.0 (ref.)	–	2.5 ±2.3
Total Nordic JIA cohort	377	3.2 ±1.5	99 (26)	2.6 (1.4–4.9)	0.002	2.7 (1.4–5.1)	0.002	3.8 ±2.7^h^
Sleep quality^e^								
Good sleep, PSQI ≤5	213	2.7 ±1.2	32 (15)	1.0 (ref.)	–	1.0 (ref.)	–	2.8 ±2.5
Poor sleep, PSQI > 5	158	3.9 ±1.4	66 (42)	4.1 (2.5–6.6)	< 0.001	3.7 (2.2–6.1)	< 0.001	5.3 ±2.4
VAS pain								
0	153	2.6 ±1.1	20 (13)	1.0 (ref.)	–	1.0 (ref.)	–	2.9 ±2.6^h^
> 0	217	3.7 ±1.5	79 (36)	3.8 (2.2–6.6)	< 0.001	3.7 (2.1–6.5)	< 0.001	4.5 ±2.7
Participation in work/study								
Full	301	3.0 ±1.3	64 (21)	1.0 (ref.)	–	1.0 (ref.)	–	3.6 ±2.6^h^
Partial	33	4.0 ±1.7	15 (45)	3.1 (1.5–6.5)	0.003	2.8 (1.3–6.0)	0.008	4.1 ±2.9
No	38	4.0 ±1.8	19 (50)	3.7 (1.9–7.4)	< 0.001	3.5 (1.7–7.3)	0.001	5.1 ±3.1
SF-36								
PCS ≥40	322	3.0 ±1.3	64 (20)	1.0 (ref.)	–	1.0 (ref.)	–	3.4 ±2.6^h^
PCS < 40	55	4.7 ±1.6	35 (64)	7.1 (3.8–13.0)	< 0.001	6.2 (3.3–11.8)	< 0.001	6.2 ±2.4
MCS ≥40	310	2.9 ±1.3	58 (19)	1.0 (ref.)	–	1.0 (ref.)	–	3.3 ±2.6^h^
MCS < 40	67	4.6 ±1.5	41 (61)	6.9 (3.9–12.1)	< 0.001	7.1 (3.9–13.0)	< 0.001	6.5 ±1.9
HAQ								
=0	260	2.8 ±1.3	47 (18)	1.0 (ref.)	–	1.0 (ref.)	–	3.2 ±2.5^h^
> 0	110	4.1 ±1.6	52 (47)	4.1 (2.5–6.6)	< 0.001	3.7 (2.2–6.1)	< 0.001	5.4 ±2.7
Disease status^f^								
Remission off med.	112	2.9 ±1.4	21 (19)	1.0 (ref.)	–	1.0 (ref.)	–	3.2 ±2.7^h^
Inactive disease	75	3.2 ±1.5	22 (29)	1.8 (0.9–3.6)	0.09	2.0 (1.0–4.1)	0.06	3.7 ±2.7
Active disease	121	3.6 ±1.6	43 (36)	2.4 (1.3–4.4)	0.005	2.2 (1.2–4.2)	0.01	4.4 ±2.8
Not ascertained^g^	61	3.1 ±1.3	12 (20)	1.0 (0.5–2.3)	0.9	1.0 (0.4–2.2)	1.0	4.3 ±2.5

*JIA* juvenile idiopathic arthritis, *No.* numbers, *SD* standard deviation, *OR* odds ratio for Fatigue Severity Scale ≥4, *CI* confidence interval, *ref.* reference, *VAS pain* self-reported pain measured on a 21-numbered circle visual analogue scale (0 = no pain, 10 = maximum pain), *SF-36* 36-Item Short Form Health Survey, 0–100 (< 40 poor health), *PCS* physical component summary, *MCS* mental component summary, *HAQ* Health Assessment Questionnaire, 0–3 (0 = lowest, 3 = highest)

^a^Fatigue measured with Fatigue Severity Scale global score, 1–7 (1 = lowest, 7 = highest)

^b^Fatigue Severity Scale ≥4

^c^Adjusted for age and sex

^d^Fatigue Severity Scale visual analogue scale, 21-numbered circle VAS (0 = no fatigue, 10 = maximum fatigue)

^e^Sleep quality measured with Pittsburgh Sleep Quality Index global score (PSQI), 0–21 (0 = best, 21 = worst)

^f^According to the definition by Wallace et al.; Remission off med. = remission off medication for ≥12 months. Inactive disease = inactive disease on medication less than 6 months or inactive disease off medication less than 12 months or remission on medication (inactive disease on medication for more than 6 months). Active disease = flare or continuous active disease

^g^Not ascertained = participated only in a telephone interview

^h^1 of 377 participants did not fill in VAS fatigue

### Patient-reported outcome measures (PROMs), HRQoL and fatigue

Participants with poor sleep, pain or physical disability reported higher mean fatigue and two- to threefold more severe fatigue, compared to participants with good sleep, no pain or no physical disability, who reported fatigue scores similar to controls (Table 2). More than twice as many young adults with no or partial participation in school/work reported severe fatigue, compared to those in fulltime school/work. The highest mean fatigue was found in participants reporting poor physical health (PCS < 40), and severe fatigue was considerably more frequent among participants with PCS < 40, compared to PCS ≥40 (64% versus 20%, adjusted OR 6.2, *p* < 0.001). Similar results were observed in those with poor mental health. Sleep quality was poor, but similar, among participants with JIA and controls, but in participants with JIA, fatigue, pain and physical disability were associated with considerably poorer sleep compared to participants without these challenges (Supplementary Table S2).

### Disease status and fatigue

**Fig. 1:**
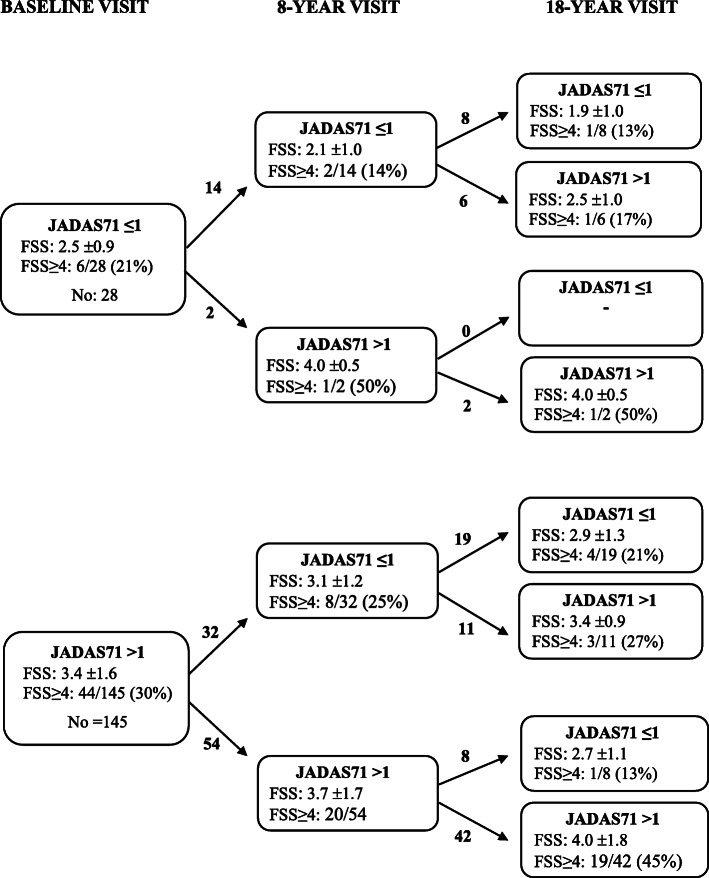
Fatigue scores measured with Fatigue Severity Scale (FSS) global score, 1–7 (1 = lowest, 7 = highest) at the 18-year follow-up in the Nordic JIA study, and compared with disease activity at baseline, 8-year and 18-year follow-up. Disease activity is measured with JADAS71, based on evaluation of 71 joints, score ≤ 1 indicates inactive disease and > 1 indicates active disease. The FSS values are mean ±SD. Severe fatigue is defined as FSS≥4, the values are number/total number (%). JIA = juvenile idiopathic arthritis

Participants with active disease reported higher mean (±SD) fatigue, compared to those in remission off medication, 3.6 (±1.6) versus 2.9 (±1.4) (Table 2). Severe fatigue was also more frequent among individuals with active disease compared to those in remission off medication, 36% versus 19% (adjusted OR 2.2 (95% CI 1.2–4.2), *p* = 0.01). The association between disease status and fatigue were maintained when analyzing single items taken from criteria for inactive disease and remission, such as ESR and physician-reported items, and not only for the patient-reported items (results not shown) [34, 35]. Participants with inactive disease both at 18-year follow-up and at baseline and/or 8-year follow-up, had the lowest fatigue score and those with active disease at all three time-points had the highest fatigue scores (Fig. 1 and Supplementary Table S4).

### Medication and fatigue

During the course of disease, 30% had been treated with biologics, 61% with synthetic DMARDs, and 43% with systemic steroids for shorter or longer duration (Table 3). Participants who had at any time been treated with biologics reported higher mean fatigue, and had higher prevalence of severe fatigue, 37% versus 22% (adjusted OR 2.3 (95% CI 1.4–3.8), *p* = 0.001), compared to those who had never used biologics. Results were similar for participants who had ever been treated with DMARDs or systemic steroids, versus no DMARDs or no systemic steroids. Also, participants with ongoing treatment with biologics or DMARDs at the 18-year follow-up had higher mean fatigue, and higher prevalence of severe fatigue, compared to participants without such treatment (Supplementary Table S3).

**Table 3:** Association between medication ever and fatigue at 18-year follow-up in the Nordic JIA cohort

			Severe fatigue^b^				
Medication ever used during disease course	No. assessed	Fatigue^a^ mean ±SD	No. (%)	OR (95% CI) crude	*p*-value	OR (95% CI) adjusted^c^	*p*-value
DMARDs (± biologics)							
NO	146	2.8 ±1.3	27 (18)	1.0 (ref.)	–	1.0 (ref.)	–
YES	231	3.4 ±1.5	72 (31)	2.0 (1.2–3.3)	0.007	2.1 (1.3–3.6)	0.004
Biologics (± DMARDs)							
NO	264	3.0 ±1.4	57 (22)	1.0 (ref.)	–	1.0 (ref.)	–
YES	113	3.6 ±1.6	42 (37)	2.1 (1.3–3.5)	0.002	2.3 (1.4–3.8)	0.001
Systemic steroids^d^							
NO	207	3.0 ±1.4	44 (21)	1.0 (ref.)	–	1.0 (ref.)	–
YES	159	3.5 ±1.6	52 (33)	1.8 (1.1–2.9)	0.01	1.8 (1.1–3.0)	0.02

*JIA* juvenile idiopathic arthritis, *No.* numbers, *SD* standard deviation, *OR* odds ratio for Fatigue Severity Scale ≥4, *CI* confidence interval, *DMARDs* disease-modifying anti-rheumatic drugs, included methotrexate, azathioprine, hydroxychloroquine, leflunomide, sulfasalazine and mycophenolate mofetil, *biologics* biologic drugs, included etanercept, infliximab, adalimumab, certolizumab, golimumab, rituximab, abatacept, anakinra, canakinumab, rilonacept and tocilizumab, *Systemic steroids* corticosteroids, oral or intravenous

^a^Fatigue measured with Fatigue Severity Scale global score, 1–7 (1 = lowest, 7 = highest)

^b^Fatigue Severity Scale ≥4

^c^Adjusted for age and sex

^d^11 participants with missing information on systemic steroids

### Predictive variables at baseline and 8-year

The association between variables at the baseline or 8-year visit and fatigue at the 18-year follow-up are shown in Table 4. We found no association between age at onset or anti-nuclear antibodies (ANA) and 18-year fatigue scores. Participants with human leucocyte antigen B27 (HLA-B27) or > 4 cumulative active joints at baseline tended to report higher mean fatigue and more severe fatigue at the 18-year follow-up. Among baseline variables, female sex and diagnostic delay ≥6 months were predictors of higher mean fatigue and severe fatigue at the 18-year follow-up. A higher proportion of participants reporting pain at baseline, reported severe fatigue at the 18-year follow-up, compared to those reporting no pain at baseline (31% versus 16%, respectively). Similar results were observed in those reporting pain at the 8-year visit. Participants with functional disability (CHQ PhS < 40), poor health (CHAQ/HAQ > 0), active disease (JADAS71 > 1) or previous/ongoing use of DMARDs/biologics at the 8-year visit, reported higher mean fatigue and more severe fatigue at the 18-year follow-up.

**Table 4:** Association between baseline and 8-year variables and fatigue at 18-year follow-up

	18-year follow-up						
			Severe fatigue^b^				
	No. assessed	Fatigue^a^ mean ±SD	No. (%)	OR (95% CI) crude	*p*-value	OR (95% CI) adjusted^c^	*p*-value
**Baseline variables**							
Sex							
Male	106	2.5 ±1.1	10 (9)	1.0 (ref.)	–	1.0 (ref.)	–
Female	271	3.5 ±1.5	89 (33)	4.7 (2.3–9.4)	< 0.001	4.6 (2.3–9.3)	< 0.001
Onset age							
< 6y	196	3.1 ±1.4	48 (24)	1.0 (ref.)	–	1.0 (ref.)	–
≥6y	179	3.3 ±1.5	50 (28)	1.2 (0.8–1.9)	0.5	1.1 (0.7–1.8)	0.6
Diagnostic delay							
< 6 months	304	3.1 ±1.4	72 (24)	1.0 (ref.)	–	1.0 (ref.)	–
≥6 months	41	3.9 ±1.7	18 (44)	2.5 (1.3–4.9)	0.007	2.9 (1.4–5.9)	0.004
ANA							
Negative	146	3.2 ±1.5	37 (25)	1.0 (ref.)	–	1.0 (ref.)	–
Positive	62	3.3 ±1.5	16 (26)	1.0 (0.5–2.0)	0.9	0.9 (0.5–1.9)	0.9
HLA-B27							
Negative	217	3.2 ±1.5	55 (25)	1.0 (ref.)	–	1.0 (ref.)	–
Positive	60	3.4 ±1.6	20 (33)	1.5 (0.8–2.7)	0.2	1.7 (0.9–3.3)	0.1
VAS pain^d^							
=0	50	2.5 ±1.3	8 (16)	1.0 (ref.)	–	1.0 (ref.)	–
> 0	161	3.4 ±1.6	50 (31)	2.4 (1.0–5.4)	0.04	2.2 (0.9–5.1)	0.08
Cumulative active joints							
≤4 joints	232	3.1 ±1.5	53 (23)	1.0 (ref.)	–	1.0 (ref.)	–
> 4 joints	135	3.4 ±1.5	43 (32)	1.6 (1.0–2.5)	0.06	1.5 (0.9–2.5)	0.1
**Variables at 8-year visit**							
VAS pain							
=0	139	2.7 ±1.4	25 (18)	1.0 (ref.)	–	1.0 (ref.)	–
> 0	151	3.5 ±1.5	51 (34)	2.3 (1.3–4.0)	0.003	2.3 (1.3–4.0)	0.005
CHQ PhS							
≥40	133	2.9 ±1.3	29 (22)	1.0 (ref.)	–	1.0 (ref.)	–
< 40	32	4.0 ±1.8	16 (50)	3.6 (1.6–8.0)	0.002	3.7 (1.5–8.9)	0.004
CHQ PsS							
≥40	152	3.1 ±1.4	39 (26)	1.0 (ref.)	–	1.0 (ref.)	–
< 40	13	3.7 ±1.9	6 (46)	2.5 (0.8–7.8)	0.1	2.6 (0.7–9.3)	0.1
CHAQ/HAQ							
=0	193	2.9 ±1.4	38 (20)	1.0 (ref.)	–	1.0 (ref.)	–
> 0	104	3.7 ±1.6	40 (38)	2.5 (1.5–4.3)	0.001	2.3 (1.3–3.9)	0.004
JADAS71							
≤1	96	2.8 ±1.2	16 (17)	1.0 (ref.)	–	1.0 (ref.)	–
> 1	97	3.7 ±1.6	37 (38)	3.1 (1.6–6.1)	0.001	2.8 (1.4–5.8)	0.004
DMARDs/biologics^e^							
No	152	2.8 ±1.3	30 (20)	1.0 (ref.)	–	1.0 (ref.)	–
Yes	215	3.3 ±1.4	66 (31)	1.8 (1.1–3.0)	0.02	2.0 (1.2–3.3)	0.009

*JIA* juvenile idiopathic arthritis, *No.* numbers, *SD* standard deviation, *OR* odds ratio for Fatigue Severity Scale ≥4, *CI* confidence interval, *ref.* reference, *ANA* anti-nuclear antibody, measured twice at least 3 months apart, *HLA-B27* human leucocyte antigen B27, *VAS pain* self-reported pain measured on a 21-numbered circle visual analogue scale (0 = no pain, 10 = maximum pain), *CHQ* Child Health Questionnaire, 0–100 (< 40 poor health), *PhS* physical summary score, *PsS* psychosocial summary score, *CHAQ* Childhood Health Assessment Questionnaire, age < 18 years, *HAQ* Health Assessment Questionnaire, age ≥18 years, 0–3 (0 = lowest, 3 = highest), *JADAS71* juvenile arthritis disease activity score based on evaluation of 71 joints, score ≤ 1 indicates inactive disease according to Consolaro et al., *DMARDs* disease-modifying anti-rheumatic drugs; included methotrexate, azathioprine, hydroxychloroquine, leflunomide, sulfasalazine and mycophenolate mofetil, *biologics* biologics drugs; included etanercept, infliximab, adalimumab, certolizumab, golimumab, rituximab, abatacept, anakinra, canakinumab, rilonacept and tocilizumab

^a^Fatigue measured with Fatigue Severity Scale global score, 1–7 (1 = lowest, 7 = highest)

^b^Fatigue Severity Scale ≥4

^c^Adjusted for age and sex

^d^All Finnish participants excluded due to missing pain scores at baseline

^e^Use of DMARDs and/or biologics from baseline to 8-year visit

## Discussion

In our longitudinal Nordic JIA cohort, we found both more fatigue and more severe fatigue among young adults with self-reported sleep problems, pain, poor health, reduced participation in school/work, physical disability, active disease, or previous or ongoing DMARDs/biologics/systemic steroids. In contrast, participants with no sleep problems, no pain, no physical disability, with self-reported good health, or in remission off medication, had fatigue scores similar to controls. Active disease at all three time points (baseline, 8-year and 18-year follow-up) was associated with higher mean fatigue score and higher percentage of severe fatigue than disease courses characterized by periods of inactive disease. Baseline variables such as female sex and diagnostic delay ≥6 months predicted fatigue at the 18-year follow-up. In addition, pain, self-reported poor health, active disease and previous/ongoing use of DMARDs/biologics at the 8-year visit predicted severe fatigue at the 18-year follow-up.

The strength of our study is the population-based, longitudinal and non-selected design, which enabled us to evaluate long-term outcome with validated multidimensional measurements. The proportion of participants lost to follow-up or with missing fatigue data (26%) is lower than in other longitudinal studies [38–40]. The novelty in looking at patient-reported fatigue compared to a control group, is a major strength. Some limitations must be mentioned that may affect the validity of our results. We performed several statistical tests throughout the present study. With increasing number of tests, the chance of false positive findings increases. Thus, we cannot exclude the possibility of some chance findings, and the results, including the *p*-values, should therefore be interpreted with caution. With 20 independent tests, the alpha level according to a Bonferroni correction would change from 0.05 to 0.0025. Still, many outcomes were related (not independent) and pointed in the same direction. More males were excluded due to missing fatigue scores. This may have skewed the results towards increased fatigue, because females report more fatigue compared to males [41, 42]. However, when we analyzed females and males separately, we found the same distinct patterns in both groups. Since fatigue was measured only at the 18-year follow-up, we had no possibility to compare fatigue scores at various timepoint during the course of disease. A limitation is also that our database contains information of ongoing medication and medication used during the course of disease, but no details on total duration of the different drugs or cumulative doses. Another limitation is the validity of data collected in telephone interviews. However, we found no significant differences in fatigue scores between participants giving telephone interview, compared to those with a clinical visit. Finally, the impact of socioeconomic status on fatigue scores was not included in the analysis.

To our knowledge, only four studies in the last two decades have focused on fatigue in JIA and controls with long-term follow-up. Three of these are cross-sectional studies of children with considerably shorter disease duration than ours, two used “historic” controls [8, 43, 44]. In agreement with our results, they reported more fatigue among JIA than controls, although, values of fatigue are difficult to compare, because different measurements are validated for children and adults. The only other prospective long-term study on fatigue in young adults with JIA and controls, is a Norwegian study that focused on general health from childhood to adulthood [45]. Unlike our study, no clinical examinations were performed, and no comparison with remission status could be presented, but participants filled in questionnaires, including VAS fatigue. In accordance with our study, this study reported approximately twofold higher proportions of moderate/severe fatigue among participants with JIA compared to controls. However, the actual numbers are not comparable as different fatigue measurements and cut-offs were used. In contrast, a Brazilian study reported no significant differences in fatigue between children with JIA and controls [46]. This study gave limited descriptive information, making further comparisons difficult.

A few studies have assessed fatigue in adults with JIA without comparison to controls. One study from a German biologic register, had results corresponding to ours with 25% reporting moderate to severe fatigue [4]. Although their participants were skewed into the severe end of the spectrum of JIA whereas we included the full disease spectrum, about 2/3 had low or no disease activity. In a long-term follow-up performed by Østlie et al., 60% of the participants with JIA reported fatigue to a certain extent, but the proportion of severe fatigue was not explored [10]. Despite small numbers, all studies of fatigue in JIA conclude that fatigue is a prominent symptom even in adult life [4, 10, 45].

Consistent with our results, several studies of JIA have shown associations between PROMs and fatigue, and that fatigue is associated to pain, sleep quality, physical and psychosocial health [4, 10, 14, 43, 45]. The correlation between fatigue and different PROMs is also one of the conclusions in a systematic review by Armbrust et al. [7]. PROMs are subjective, as they mirror the patient’s own experience, and they are related to each other, and accordingly often difficult to separate. This inter-relationship makes conclusions on causality difficult. However, in our study we found that participants with JIA as a group reported similar poor sleep quality as the control group. This underlines the importance of comparison to a control group, indicating that not all negative PROMs have to be disease-related.

Similar to studies of JIA, few longitudinal studies of RA and fatigue have included controls, and only one during the last two decades, reporting higher mean fatigue (measured with FSS) among participants with RA than among controls [47]. In addition, two older studies from the 1990s reported similar results [48, 49].

Studies of both JIA and RA have shown an inconsistent relationship between fatigue and disease activity. In our study, participants with active disease reported more fatigue compared to those in remission off medication or inactive disease. These results are in agreement with several other studies of JIA [14, 46, 50], and consistent with a large international study of about 10,000 RA participants [51]. Other studies found no significant association between disease activity in JIA and fatigue. Two studies of children/adolescents with JIA, showed more fatigue among those with active compared to inactive disease, but after adjustment for pain, the association was substantially attenuated [8, 13]. We have not adjusted for pain, because we consider pain to be a potential mediator and not a confounder, since pain cannot cause disease activity. If we adjusted for pain, our results were also attenuated. This does not imply that pain is a confounder but suggests that pain may be a mediator on the causal pathway between JIA and fatigue. When analyzing single objective and physician-reported items of disease status, the association between disease activity and fatigue remained unchanged, supporting that the association is not based on only patient-reported information. Furthermore, the fact that individuals with active disease at all three time points (baseline, 8-year and 18-year follow-up) had the highest fatigue scores, highlights the negative effect of long-term disease activity on the development of fatigue.

Studies of associations between medication and fatigue in adults with JIA are few. Unlike our study, three studies from the Netherlands and United States, found no significant correlation between fatigue and medication in children with JIA [6, 8, 44]. In contrast, a Canadian study reported more fatigue among individuals using DMARDs, which is consistent with our results [14]. For RA, studies on associations between medication and fatigue, showed different results [15, 52]. It is difficult to differentiate the effect of long-term disease activity and medication, especially in longitudinal population-based studies. It may be that duration of active disease and disease severity more than medication itself, explain the association between medication and fatigue in our study. Furthermore, the impact of medication must be interpreted with care, because we use a dichotomous model since we have limited information about duration of treatment or if the medication was used once or repeatedly.

Early predictors of long-term outcome in JIA, have been difficult to identify. Some have studied early predictors for negative outcome, like disease activity and reduced HRQoL [10, 32], but studies predicting fatigue as a long-term outcome measure in adults with JIA seem to be lacking. It is well-known that female sex is associated with more fatigue [41, 42]. In addition, the number of active joints, and pain are known negative outcome predictors [45]. It is more surprising that diagnostic delay showed a clear association to more severe fatigue. One may speculate that delayed treatment and thus longer duration of active disease or no treatment within the “window of opportunities” may be some of the explanation.

The results of our study have potential implications for clinical practice. Several patient/physician-reported variables both at baseline and during disease course predicted fatigue in a long-term perspective. Additionally, fatigue was strongly associated with other negative disease outcomes at the 18-year follow-up. On the positive side, young adults with JIA in clinical remission off medication and those who reported good health, had fatigue scores similar to controls. Fatigue has previously been stated by the patients as one of the most important outcomes of their disease [16, 53]. Taken together, these results indicate that fatigue is a clinical meaningful outcome factor, and fatigue measurement should be included in direct clinical judgements, in clinical trials, and in composite outcome measurements in JIA, and further, may be an essential part in future treatment strategies.

## Conclusions

In conclusion, fatigue is a prominent symptom and 26% reported severe fatigue in this 18-year follow-up study of young adults with JIA, compared to 12% in the control group. We found a consistent and higher fatigue burden among participants with poor sleep, pain, self-reported health problems, active disease, or previous/ongoing use of DMARDs/biologics, compared to participants without these challenges and compared to controls. Several patient/physician-reported variables predicted fatigue 18 years after disease onset. We suggest that fatigue should be measured regularly in both pediatric and adult rheumatology clinics and in future research.

## Supplementary Information

**Additional file 1: Figure S1.** The distribution of fatigue scores at the 18-year follow-up of participants with juvenile idiopathic arthritis (JIA) in the Nordic JIA study and in the Norwegian control group. Fatigue is measured with Fatigue Severity Scale (FSS) global score, 1–7 (1 = lowest, 7 = highest). The dot-plot illustrating the distribution of fatigue scores for individual participants within each group as dots, and the group median indicated with the horizontal spiked line.

**Additional file 2: Table S1.** Fatigue score according to JIA category in the Nordic JIA cohort at 18-year follow-up. **Table S2.** Sleep quality in the Nordic JIA cohort according to clinical characteristics at 18-year follow-up. **Table S3.** Association between ongoing medication and fatigue at 18-year follow-up in the Nordic JIA cohort. **Table S4.** Association between changes in disease activity and fatigue scores in the Nordic JIA cohort.

## Data Availability

The datasets generated and/or analyzed during the current study are not publicly available for ethical reasons, as well as privacy reasons, but are available from the Nordic Study group of Pediatric Rheumatology (NoSPeR) on reasonable request.

## Abbreviations

ACR: American College of Rheumatology
ANA: Anti-nuclear antibodies
CHAQ: Childhood Health Assessment Questionnaire
CI: Confidence interval
CHQ PhS: Child Health Questionnaire Physical summary score
CHQ PsS: Child Health Questionnaire Psychosocial summary score
DMARD: Disease-modifying anti-rheumatic drugs
ESR: Erythrocyte sedimentation rate
FSS: Fatigue Severity Scale
HAQ: Health Assessment Questionnaire
HLA-B27: Human leucocyte antigen B27
HRQoL: Health-related quality of life
ILAR: International League of Associations for Rheumatology
IQR: Interquartile range
JADAS: Juvenile Arthritis Disease Activity Score
JIA: Juvenile idiopathic arthritis
MCS: SF-36 Mental component summary
OR: Odds ratio
PCS: SF-36 Physical component summary
PROMs: Patient-reported outcome measures
PSQI: Pittsburgh Sleep Quality Index
RA: Rheumatoid arthritis
SD: Standard deviation
SF-36: 36-Item Short-Form Health Survey
VAS: Visual analogue scale
