# Supplementary figures and images for: Fatigue in young adults with juvenile idiopathic arthritis 18 years after disease onset: data from the prospective Nordic JIA cohort
Source: Pediatr Rheumatol Online J. 2021 Mar 18;19:33. doi: 10.1186/s12969-021-00499-0 (PMC7976696; doi:10.1186/s12969-021-00499-0)

# Fatigue Scores at 18-year follow-up

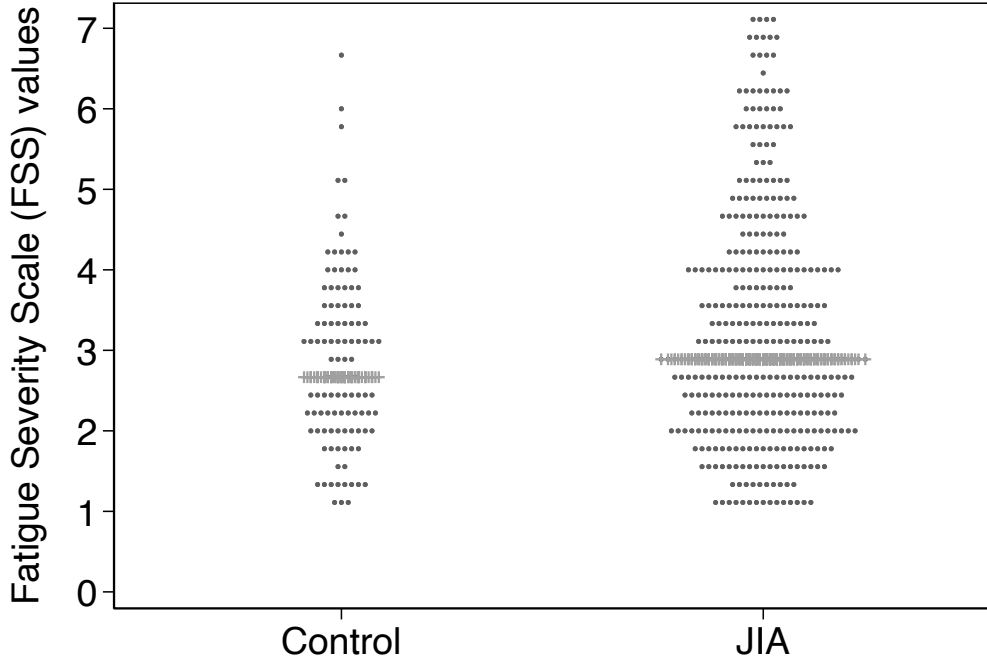

Supplement: Supplementary file 1 — Additional file 1: Figure S1. The distribution of fatigue scores at the 18-year follow-up of participants with juvenile idiopathic arthritis (JIA) in the Nordic JIA study and in the Norwegian control group. Fatigue is measured with Fatigue Severity Scale (FSS) global score, 1–7 (1 = lowest, 7 = highest). The dot-plot illustrating the distribution of fatigue scores for individual participants within each group as dots, and the group median indicated with the horizontal spiked line. [file 12969_2021_499_MOESM1_ESM.pdf]
