# Supplementary material for: Fatigue in young adults with juvenile idiopathic arthritis 18 years after disease onset: data from the prospective Nordic JIA cohort
Source: Pediatr Rheumatol Online J. 2021 Mar 18;19:33. doi: 10.1186/s12969-021-00499-0 (PMC7976696; doi:10.1186/s12969-021-00499-0)
Supplement: Supplementary file 2 — Additional file 2: Table S1. Fatigue score according to JIA category in the Nordic JIA cohort at 18-year follow-up. Table S2. Sleep quality in the Nordic JIA cohort according to clinical characteristics at 18-year follow-up. Table S3. Association between ongoing medication and fatigue at 18-year follow-up in the Nordic JIA cohort. Table S4. Association between changes in disease activity and fatigue scores in the Nordic JIA cohort. [file 12969_2021_499_MOESM2_ESM.zip › Supplementary NEW Table S2 Sleep_Pediatric Rheumatology _Proof.pdf]

Supplementary Table S2. Sleep quality in the Nordic JIA cohort according to clinical characteristics at 18-year follow-up

	No. assessed	Sleep quality[a] mean ±SD	Poor sleep[b]				
			No. (%)	OR (95% CI) crude	p-value	OR (95% CI) adjusted[c]	p-value
Norwegian controls	109	5.9 ±3.0	50 (46)	1.0 (ref.)	-	1.0 (ref.)	-
Total Nordic JIA cohort	371	5.7 ±3.5	158 (43)	0.9 (0.6-1.3)	0.5	0.8 (0.5-1.3)	0.5
Male	103	4.9 ±2.9	33 (32)	1.0 (ref.)	-	1.0 (ref.)	-
Female	268	6.0 ±3.7	125 (47)	1.9 (1.1-3.0)	0.01	1.8 (1.1-3.0)	0.01
Fatigue[d]							
Not severe, FSS <4	273	4.8 ±2.8	92 (34)	1.0 (ref.)	-	1.0 (ref.)	-
Severe, FSS ≥4	98	8.2 ±4.0	66 (67)	4.1 (2.5-6.6)	<0.001	3.7 (2.2-6.1)	<0.001
VAS pain							
=0	151	4.4 ±2.9	242 (28)	1.0 (ref.)	-	1.0 (ref.)	-
>0	213	6.7 ±3.6	115 (54)	3.0 (1.9-4.8)	<0.001	2.9 (1.9-4.6)	<0.001
Participation in work/studies							
Full	297	5.3 ±3.2	113 (38)	1.0 (ref.)	-	1.0 (ref.)	-
Partial	32	6.9 ±4.3	19 (59)	2.4 (1.1-5.0)	0.02	2.3 (1.1-4.8)	0.03
No	37	8.0 ±4.6	24 (65)	3.0 (1.5-6.1)	0.003	2.9 (1.4-5.9)	0.004
SF-36							
PCS ≥40	316	5.1 ±3.2	113 (36)	1.0 (ref.)	-	1.0 (ref.)	-
PCS <40	55	9.0 ±3.7	45 (82)	8.1 (3.9-16.7)	<0.001	7.6 (3.6-15.7)	<0.001
MCS ≥40	305	5.0 ±3.0	107 (35)	1.0 (ref.)	-	1.0 (ref.)	-
MCS <40	66	9.0 ±4.0	51 (77)	6.3 (3.4-11.7)	<0.001	6.2 (3.3-11.6)	<0.001
HAQ							
=0	256	4.9 ±2.9	88 (34)	1.0 (ref.)	-	1.0 (ref.)	-
>0	108	7.7 ±4.0	69 (64)	3.4 (2.1-5.4)	<0.001	3.2 (2.0-5.1)	<0.001
Disease status[e]							
Remission off med.	111	5.0 ±3.6	39 (35)	1.0 (ref.)	-	1.0 (ref.)	-
Inactive disease	72	5.8 ±3.8	26 (36)	1.0 (0.6-1.9)	0.9	1.1 (0.6-2.0)	0.9
Active disease	120	6.4 ±3.5	62 (52)	2.0 (1.2-3.4)	0.01	1.9 (1.1-3.2)	0.02
Not ascertained[f]	60	5.5 ±3.0	28 (47)	1.6 (0.9-3.1)	0.1	1.6 (0.8-3.0)	0.2

JIA = juvenile idiopathic arthritis; No. = numbers; SD = standard deviation; OR = odds ratio for Pittsburgh Sleep Quality Index global score >5; CI = confidence interval; ref. = reference; VAS pain = self-reported pain measured

on a 21-numbered circle visual analogue scale (0 = no pain, 10 = maximum pain); SF-36 = 36-Item Short Form Health Survey, 0-100 (<40 poor health), PCS = physical component summary, MCS = mental component summary; HAQ = Health Assessment Questionnaire, 0-3 (0 = lowest, 3 = highest).

[a] Sleep quality measured with Pittsburgh Sleep Quality Index global score, 0-21 (0 = best, 21 = worst).
[b] Pittsburgh Sleep Quality Index global score >5.
[c] Adjusted for age and sex.
[d] Fatigue measured with Fatigue Severity Scale global score (FSS), 1-7 (1 = lowest, 7 = highest).
[e] According to the definition by Wallace et al.; Remission off med. = remission off medication for ≥12 months. Inactive disease = inactive disease on medication less than 6 months or inactive disease off medication less than 12 months or remission on medication (inactive disease on medication for more than 6 months). Active disease = flare or continuous active disease.
[f] Not ascertained = participated only in a telephone interview.
