# Supplementary material for: Fatigue in young adults with juvenile idiopathic arthritis 18 years after disease onset: data from the prospective Nordic JIA cohort
Source: Pediatr Rheumatol Online J. 2021 Mar 18;19:33. doi: 10.1186/s12969-021-00499-0 (PMC7976696; doi:10.1186/s12969-021-00499-0)
Supplement: Supplementary file 2 — Additional file 2: Table S1. Fatigue score according to JIA category in the Nordic JIA cohort at 18-year follow-up. Table S2. Sleep quality in the Nordic JIA cohort according to clinical characteristics at 18-year follow-up. Table S3. Association between ongoing medication and fatigue at 18-year follow-up in the Nordic JIA cohort. Table S4. Association between changes in disease activity and fatigue scores in the Nordic JIA cohort. [file 12969_2021_499_MOESM2_ESM.zip › Supplementary NEW Table S3 MedicationOngoing_Fatigue_Pediatric Rheumatology _Proof.pdf]

Supplementary Table S3. Association between ongoing medication and fatigue at 18-year follow-up in the Nordic JIA cohort

			Severe fatigue[b]				
Medication ongoing at 18-year follow-up	No. assessed	Fatigue[a] mean ±SD	No. (%)	OR (95% CI) crude	p-value	OR (95% CI) adjusted[c]	p-value
DMARDs (± biologics)							
NO	294	3.1 ±1.4	70 (24)	1.0 (ref.)	-	1.0 (ref.)	-
YES	83	3.7 ±1.5	29 (35)	1.7 (1.0-2.9)	0.04	1.5 (0.9-2.6)	0.1
Biologics (± DMARDs)							
NO	297	3.1 ±1.4	69 (23)	1.0 (ref.)	-	1.0 (ref.)	-
YES	80	3.6 ±1.6	30 (38)	2.0 (1.2-3.4)	0.01	2.3 (1.3-4.0)	0.004

JIA = juvenile idiopathic arthritis; No. = numbers; SD = standard deviation; OR = odds ratio for Fatigue Severity Scale ≥4; CI = confidence interval; DMARDs = disease-modifying anti-rheumatic drugs, included methotrexate, azathioprine, hydroxychloroquine, leflunomide, sulfasalazine and mycophenolate mofetil; biologics = biologic drugs, included etanercept, infliximab, adalimumab, certolizumab, golimumab, rituximab, abatacept, anakinra, canakinumab, rilonacept and tocilizumab.

[a] Fatigue measured with Fatigue Severity Scale global score, 1-7 (1 = lowest, 7 = highest).

[b] Fatigue Severity Scale ≥4.

[c] Adjusted for age and sex.
