# Supplementary material for: Fatigue in young adults with juvenile idiopathic arthritis 18 years after disease onset: data from the prospective Nordic JIA cohort
Source: Pediatr Rheumatol Online J. 2021 Mar 18;19:33. doi: 10.1186/s12969-021-00499-0 (PMC7976696; doi:10.1186/s12969-021-00499-0)
Supplement: Supplementary file 2 — Additional file 2: Table S1. Fatigue score according to JIA category in the Nordic JIA cohort at 18-year follow-up. Table S2. Sleep quality in the Nordic JIA cohort according to clinical characteristics at 18-year follow-up. Table S3. Association between ongoing medication and fatigue at 18-year follow-up in the Nordic JIA cohort. Table S4. Association between changes in disease activity and fatigue scores in the Nordic JIA cohort. [file 12969_2021_499_MOESM2_ESM.zip › Supplementary NEW Table S4_Pediatric Rheumatology _Proof.pdf]

Supplementary Table S4. Association between disease activity and fatigue scores in the Nordic JIA cohort at 18-years follow-up, compared to disease activity at baseline and 8-year visit

	18-year follow-up				
	Disease activity[a]	Fatigue[b] mean ±SD	Severe fatigue[c] no (%)	OR (95% CI)[d]	p-value
**Baseline visit**					
JADAS71 ≤1 No 28	JADAS71 ≤1	1.8 ±0.9	1/11 (9%)	1.0 (ref.)	-
	JADAS71 >1	2.7 ± 1.2	3/12 (25%)	3.3 (0.3-38.1)	0.3
JADAS71 >1 No 145	JADAS71 ≤1	2.8 ±1.3	8/43 (19%)	1.0 (ref.)	-
	JADAS71 >1	3.7 ±1.6	31/80 (39%)	2.8 (1.1-6.7)	0.03
**8-year visit**					
JADAS71 ≤1 No 96	JADAS71 ≤1	2.6 ±1.3	8/56 (14%)	1.0 (ref.)	-
	JADAS71 >1	2.9 ± 1.2	6/30 (20%)	1.5 (0.5-4.8)	0.5
JADAS71 >1 No 97	JADAS71 ≤1	3.0 ±1.0	5/26 (19%)	1.0 (ref.)	-
	JADAS71 >1	4.1 ±1.7	30/61 (49%)	4.1 (1.4-12.2)	0.01

JIA = juvenile idiopathic arthritis; No. = numbers; SD = standard deviation; OR = odds ratio for Fatigue Severity Scale ≥4; CI = confidence interval; ref. = reference; JADAS71 = juvenile arthritis disease activity score.

[a] Disease activity measured with JADAS71, based on evaluation of 71 joints, score ≤1 indicates inactive disease according to Consolaro et al., 2011.

[b] Fatigue measured with Fatigue Severity Scale global score, 1-7 (1 = lowest, 7 = highest).

[c] Fatigue Severity Scale ≥4.

[d] Crude OR.
