# Supplementary material for: Fatigue in young adults with juvenile idiopathic arthritis 18 years after disease onset: data from the prospective Nordic JIA cohort
Source: Pediatr Rheumatol Online J. 2021 Mar 18;19:33. doi: 10.1186/s12969-021-00499-0 (PMC7976696; doi:10.1186/s12969-021-00499-0)
Supplement: Supplementary file 2 — Additional file 2: Table S1. Fatigue score according to JIA category in the Nordic JIA cohort at 18-year follow-up. Table S2. Sleep quality in the Nordic JIA cohort according to clinical characteristics at 18-year follow-up. Table S3. Association between ongoing medication and fatigue at 18-year follow-up in the Nordic JIA cohort. Table S4. Association between changes in disease activity and fatigue scores in the Nordic JIA cohort. [file 12969_2021_499_MOESM2_ESM.zip › Supplementary Table S1 JIAcategory_Pediatric Rheumatology _Proof.pdf]

Supplementary Table S1. Fatigue score according to JIA category in the Nordic JIA cohort at 18-year follow-up

JIA category[a]	No. assessed	Fatigue[b] mean ±SD	Severe fatigue[c] No. (%)
Systemic	13	3.2 ±1.6	3 (23)
Oligo. persistent	98	3.0 ±1.4	25 (26)
Oligo. extended	77	3.2 ±1.6	23 (30)
Polyart. RF negative	64	3.1 ±1.3	13 (20)
Polyart. RF positive	6	4.4 ±1.4	3 (50)
Psoriatic	21	3.4 ±1.6	5 (24)
ERA	37	3.1 ±1.3	8 (22)
Undifferentiated	61	3.4 ±1.7	19 (31)

JIA = juvenile idiopathic arthritis; No. = numbers; SD = standard deviation; Oligo. = oligoarticular; Polyart. = polyarticular; RF = rheumatoid factor; ERA = enthesitis-related arthritis

[a] JIA category according to the International League of Associations for Rheumatology (ILAR) classification criteria

[b] Fatigue measured with Fatigue Severity Scale global score, 1-7 (1= lowest, 7 = highest)

[c] Fatigue Severity Scale ≥4
